# Elaborations on Corallopyronin A as a Novel Treatment Strategy Against Genital Chlamydial Infections

**DOI:** 10.3389/fmicb.2019.00943

**Published:** 2019-05-07

**Authors:** Nathalie Loeper, Simon Graspeuntner, Svea Ledig, Inga Kaufhold, Friederike Hoellen, Andrea Schiefer, Beate Henrichfreise, Kenneth Pfarr, Achim Hoerauf, Kensuke Shima, Jan Rupp

**Affiliations:** ^1^Department of Infectious Diseases and Microbiology, University of Lübeck, Lübeck, Germany; ^2^Department of Obstetrics and Gynecology, University Hospital of Schleswig-Holstein, University of Lübeck, Lübeck, Germany; ^3^Institute of Medical Microbiology, Immunology and Parasitology, University Hospital Bonn, Bonn, Germany; ^4^German Center for Infection Research (DZIF), Partner Sites Bonn-Cologne/Hamburg-Lübeck-Borstel-Riems, Lübeck, Germany; ^5^Institute of Pharmaceutical Microbiology, University Hospital Bonn, University of Bonn, Bonn, Germany

**Keywords:** Corallopyronin A, *C. trachomatis*, antibiotic treatment, novel antibiotics, RNA polymerase inhibitor

## Abstract

Ascending *Chlamydia trachomatis* infection causes functional damage to the fallopian tubes, which may lead to ectopic pregnancy and infertility in women. Treatment failures using the standard regimens of doxycycline and azithromycin have been observed. We tested the polyketide-derived α-pyrone antibiotic Corallopyronin A (CorA) that inhibits the bacterial DNA dependent RNA polymerase and has strong activity against various extracellular and some intracellular bacteria. Extensive testing in cell culture infection models and in an *ex vivo* human fallopian tube model under different oxygen concentrations was performed to assess the anti-chlamydial efficacy of CorA at physiological conditions. CorA showed high efficacy against *C. trachomatis* (MIC_N/H_: 0.5 μg/mL for serovar D and L2), *C. muridarum* (MIC_N/H_: 0.5 μg/mL), and *C. pneumoniae* (MIC_N/H_: 1 μg/mL) under normoxic (N) and hypoxic (H) conditions. Recoverable inclusion forming units were significantly lower already at 0.25 μg/mL for all tested chlamydiae. CorA at a concentration of 1 μg/mL was also effective against already established *C. trachomatis* and *C. pneumoniae* infections (up to 24 h.p.i.) in epithelial cells, while efficacy against *C. muridarum* was limited to earlier time points. A preliminary study using a *C. muridarum* genital infection model revealed corresponding limitations in the efficacy. Importantly, in an *ex vivo* human fallopian tube model, the growth of *C. trachomatis* was significantly inhibited by CorA at concentrations of 1–2 μg/mL under normoxic and hypoxic conditions. The overall high efficacies of CorA against *C. trachomatis* in cell culture and an *ex vivo* human fallopian tube model under physiological oxygen concentrations qualifies this drug as a candidate that should be further investigated.

## Introduction

Infections with *Chlamydia trachomatis* are a major health concern worldwide with more than 100 million new cases per annum (Senior, 2012; Newman et al., 2015). Recurrent infection with *C. trachomatis* is common and mediates several upper genital tract sequelae including pelvic inflammatory disease (PID), ectopic pregnancy, and infertility in women (Workowski et al., 2015). Some women diagnosed with uncomplicated cervical infection already have a subclinical infection of the upper reproductive tract including the uterus, fallopian tubes, and the ovaries (Workowski et al., 2015).

While the current recommended treatment strategies against urogenital *C. trachomatis* infections are either a 7-day course of doxycycline or a single dose of azithromycin (Workowski et al., 2015), recent reports suggest that eradication rates using azithromycin are inferior to doxycycline (Geisler et al., 2015). Treatment failures, defined as recurrence of the pathogen after so called efficient antibiotic treatment, are observed in up to 8% of the patients (Horner, 2006).

According to the above mentioned observations, we and others could previously experimentally link impaired efficacies of first-line antimicrobials to low oxygen concentrations (0.5–5.5% O_2_) (Shima et al., 2011, 2013; Schaffer and Taylor, 2015; Matsuo et al., 2019), which are observed in the human female genital tract (Juul et al., 2007; Dietz et al., 2011). In fact, hypoxia plays a key role in physiological processes as well as pathophysiological conditions, e.g., inflammation associated with bacterial infection (Schaffer and Taylor, 2015), and might thus be one of the reasons accounting for treatment failures in chlamydial infections in patients. Therefore, alternative antimicrobials with high efficacies over a broad range of oxygen concentrations are considered a feasible solution to overcome limitations of current treatment strategies.

Along this line, we analyzed the efficacy of Corallopyronin A (CorA) on infection with *Chlamydia* spp. CorA was first isolated from *Corallococcus coralloides* (Irschik et al., 1985). It is a polyketide-derived α-pyrone antibiotic that inhibits the bacterial DNA dependent RNA polymerase (RNAP) (Mukhopadhyay et al., 2008) and thereby impedes bacterial growth. CorA’s anti-bacterial property is mediated through binding to a region outside of the bacterial RNAP active site called the switch region (Srivastava et al., 2011). The antimicrobial efficacy of CorA has been shown mainly for Gram-positive bacteria (Irschik et al., 1985). However, it also eradicates the Gram-negative intracellular bacteria *Wolbachia* (Schiefer et al., 2012) and *Orientia tsutsugamushi* (Kock et al., 2018) *in vitro* and *in vivo*. We have shown that CorA is a potent antibiotic for the eradication of rifampicin-resistant *C. trachomatis* serovar L2 *in vitro* (Shima et al., 2018).

Here, we report high activity of CorA against chlamydiae in epithelial cell lines and a human fallopian tube *ex vivo* model under normoxic and hypoxic conditions. Conflicting results in a *C. muridarum in vivo* model have to be further investigated as well as the methods for predicting and transferring antimicrobial efficacy data from the mouse infection model to the situation in human *C. trachomatis* infections.

## Materials and Methods

### Bacterial Strains and Cell Culture

*Chlamydia trachomatis* serovar D (ATCC VR-885), *C. trachomatis* serovar L2 (ATCC VR-902B), *C. muridarum* NiggII (ATCC VR-123), and *C. pneumoniae* CWL029 (ATCC VR-1310) were propagated in HeLa (ATCC CCL-2) or HEp-2 cells (ATCC CCL-23) *in vitro*. Human fallopian tubes were infected *ex vivo* with *C. trachomatis* serovar D. *C. muridarum* NiggII was used for all *in vivo* experiments for the genital infection of mice.

### Chemicals

Doxycycline was purchased from Sigma-Aldrich (D9891; St. Louis, MO, United States). Purification of CorA was performed as described in previous studies with some modification (Erol et al., 2010; Schiefer et al., 2012) for all *in vitro* and *ex vivo* experiments. For the *in vivo* experiments CorA (purity of 60% by LC/MS) was diluted in dimethyl sulfoxide (DMSO) or ethanol, correcting the mass for the purity to achieve the required concentrations. Intraperitoneal (i.p.) injection was performed by using a mixture of the DMSO-CorA solution (10%) and PBS (90%), while oral administration was given using the ethanol-CorA solution (10%) mixed with Solutol^®^ HS 15 (40%, Sigma-Aldrich) and ddH_2_O (50%).

### Determination of the Minimum Inhibitory Concentration (MIC) for Chlamydiae

A total of 5 × 10^4^ HeLa cells for *C. trachomatis* and *C. muridarum* or HEp-2 cells for *C. pneumoniae* were grown in a 24-well plate (Greiner bio-one, Frickenhausen, Germany) in RPMI1640 (Gibco/Invitrogen, Karlsruhe, Germany) with 5% FCS (Gibco/Invitrogen), non-essential amino acids (GE Healthcare Hyclone, South Logan, UT) and 2 mM glutamine (Lonza, Cologne, Germany) without antibiotics for 24 h under normoxic (20% O_2_) and hypoxic (2% O_2_, Toepffer Lab Systems, Göppingen, Germany) conditions. Afterward, cells were infected with *C. trachomatis* serovar D, *C. trachomatis* serovar L2, *C. muridarum* NiggII, or *C. pneumoniae* CWL029 with an infection rate of 0.5 (50% infected cells in controls). Centrifugation (700 × *g*, 1 h, 37°C) was used for *C. trachomatis* serovar D, *C. muridarum* NiggII, and *C. pneumoniae* CWL029 infection. In addition, 0.1 μg/mL cycloheximide were used for *C. muridarum* and *C. pneumoniae* CWL029 infection. Infected cells were incubated with or without different concentrations of CorA ranging from 0.125 to 2 μg/mL. MICs were determined by the visualization of the growth of chlamydiae after 48 h incubation. Chlamydial inclusions were visualized by immunofluorescence staining with a mouse anti-chlamydial lipopolysaccharide (LPS) antibody (kindly provided by Dr. Helmut Brade, Borstel, Germany) and a polyclonal rabbit FITC-labeled anti-mouse IgG antibody (Dako, Hamburg, Germany).

### Testing of Recoverable Chlamydiae

A total of 5 × 10^4^ HeLa for *C. trachomatis* and *C. muridarum* or HEp-2 cells for *C. pneumoniae* was seeded in 24-well plates and cultured for 24 h under normoxic and hypoxic conditions. Afterward, cells were infected with *C. trachomatis* serovar D, *C. trachomatis* serovar L2, *C. muridarum*, or *C. pneumoniae* CWL029 with an infection rate of 0.5 (50% infected cells in controls). Different concentrations of CorA ranging from 0.25 to 1.5 μg/mL were added at the time of infection. In addition, 1 μg/mL CorA was added at 6, 12, and 24 h post-infection (h.p.i.), to investigate whether an established chlamydial infection can be eradicated. At 30 (*C. trachomatis* and *C. muridarum*) or 48 (*C. pneumoniae*) h.p.i., cells were washed with medium to remove the remaining CorA and recoverable chlamydiae were determined as described (Shima et al., 2011). Chlamydial inclusions were visualized as described above.

### Application of CorA in an *in vivo* Mouse Model of *Chlamydia muridarum* Infection

Eight-week old female C57BL/6JRj mice (Janvier Labs, France) were synchronized to the same stage of estrous cycle by subcutaneous injection of 2.5 mg medroxyprogesterone acetate (Depo-Clinovir^®^, Pfizer, New York, NY, United States) per mouse. After 7 days, each mouse was vaginally infected with 10^6^ IFUs C. *muridarum*. CorA and other antibiotics were applied day 1 to 8 p.i. We used an i.p. injection with DMSO (10%) as solvent or a solvent-mixture of ethanol (10%), Solutol^®^ HS 15 (40%) and ddH_2_O for oral gavage via feeding tubes (Instech Laboratories, Plymouth Meeting, PA, United States). CorA was given in concentrations of 70 mg/kg BW (once daily) and 35 mg/kg BW (once or twice daily). Other antibiotics were given as follows: doxycycline (50 mg/kg BW i.p., once daily, or 10 mg/kg BW orally, twice daily), azithromycin (40 mg/kg BW, orally, once daily), and amoxicillin (20 mg/kg BW, orally, three times daily). One group was treated with each solvent alone as control. Vaginal swabs (Oxoid Limited, Basingstoke, United Kingdom) were collected at days 3, 7, 10, 14, 17, 21, 28, and 35 p.i. and recovery assay of *C. muridarum* was performed on HEp-2 cells to determine the bacterial burden. At 43 days post-infection, mice were euthanized and the upper and lower genital tract was macroscopically and microscopically evaluated for the formation of characteristic pathologies.

### Scoring of Pathologies

To score pathologies, hydrosalpinx at the upper genital tract were classified as follows based on their size: 0 = none, 1 = microscopically visible, 2 = visible by eye, smaller than the ovaries, 3 = same size as the ovaries, 4 = bigger than the ovaries. A stereomicroscope with a magnification ranging from 4 to 31.5 was used to assess score 1.

### Efficacy of CorA Against *C. trachomatis* Serovar D in the Human Fallopian Tube *ex vivo* Model

Preparation of human fallopian tubes was performed as described (Jerchel et al., 2012). In brief, the tissue of human fallopian tubes was collected at peripartum sterilization on maternal request during cesarean sections or during hysterectomies due to symptomatic uterine fibroids in the luteal phase. The human fallopian tubes were dissected in a Petri dish containing RPMI1640 (Gibco/Invitrogen) with 5% FCS (Gibco/Invitrogen), non-essential amino acids (GE Healthcare Hyclone), and 2 mM glutamine (Lonza) without antibiotics. After dissection, human fallopian tubes were opened carefully with a small scalpel. The human fallopian tube specimen in culture medium was infected with 5 × 10^8^ IFUs *C. trachomatis* serovar D with or without CorA for 48 h under normoxic and hypoxic conditions. At 48 h.p.i., cells were washed with medium to remove the remaining CorA and recoverable *C. trachomatis* serovar D were determined.

### Ethics Statement

Experiments with explanted human fallopian tubes were approved by the ethical committee of the University of Lübeck (09-153). Participants were informed and provided written consent. The animal experiments were revised and approved by the Ministry of Energy, Agriculture, the Environment and Rural Areas of Schleswig-Holstein [File Reference V311-7224.122(54-4/13)].

### Statistics

Data are indicated as mean ± standard deviation (SD). Statistical analysis was performed using GraphPad Prism statistical software (version 7.03). Continuous variables between two groups were evaluated utilizing paired Student’s *t*-test. When three or more groups were compared in the experiment, Sidak’s multiple comparison was used if the one-way analysis of variance showed statistical significance. Groups with nominal data were compared using Chi-Square test. *P*-values ≤ 0.05 were considered as statistically significant.

## Results

### CorA Has Equivalent MIC Values Against Chlamydiae Under Normoxia and Hypoxia

**FIGURE 1:**
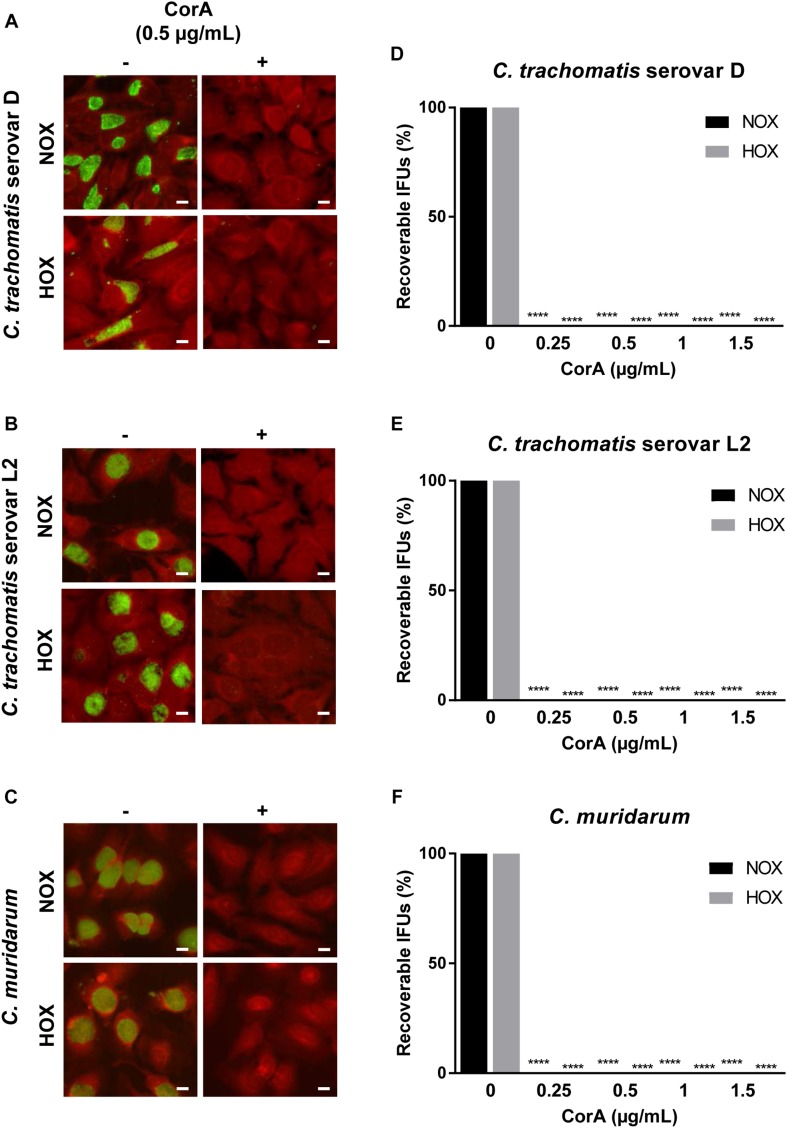
Efficacy of CorA against *Chlamydia trachomatis* and *Chlamydia muridarum* in HeLa cells under normoxia and hypoxia. The inhibition of *C. trachomatis* serovar D **(A)**, serovar L2 **(B)**, and *C. muridarum* **(C)** growth in HeLa cells upon exposure to CorA under normoxia and hypoxia. Immunofluorescence staining shows chlamydial inclusions (green) and host cells (red, scale bar = 10 μm). Recoverable IFUs were determined for *C. trachomatis* serovar D **(D)**, serovar L2 **(E)**, and *C. muridarum* **(F)**. The numbers of recoverable chlamydiae at the indicated CorA concentrations were calculated as a percentage of the untreated control under normoxia and hypoxia at 30 h post-infection (h.p.i.) (*n* = 3; mean ± SD, Sidak’s multiple comparison ^****^p ≤ 0.0001). Nox: Normoxia, Hox: Hypoxia, CorA: Corallopyronin A.

CorA is a promising drug against other intracellular pathogens, such as *Wolbachia* endobacteria and *O. tsutsugamushi* (Schiefer et al., 2012; Kock et al., 2018) and, therefore, its efficacy against *Chlamydia* spp. was determined. The MIC is defined as the minimal inhibitory concentration to prevent visible chlamydial growth in cell culture. We tested the MIC of CorA against several chlamydial species in and investigated whether the MICs of CorA differed under normoxia (20% O_2_) and hypoxia (2% O_2_). CorA had high efficacies against *C. trachomatis* serovars D and L2, *C. muridarum* and *C. pneumoniae*. The effectiveness was similar under normoxia and hypoxia (*C. trachomatis* serovar D: 0.5 μg/mL, *C. trachomatis* serovar L2: 0.5 μg/mL, *C. muridarum*: 0.5 μg/mL, and *C. pneumoniae*: 1 μg/mL) (Table 1 and Figures 1A–C).

**TABLE 1:** MIC of CorA against *Chlamydia* spp. under normoxia and hypoxia (*n* = 4).

	MIC (μg/mL)	
	Normoxia (20% O_2_	Hypoxia (2% O_2_)
*C. trachomatis* D	0.5	0.5
*C. trachomatis* L2	0.5	0.5
*C. muridarum*	0.5	0.5
*C. pneumoniae* CWL029	1	1

### CorA Treatment Significantly Reduces Infectious Progeny of Chlamydiae Under Normoxia and Hypoxia

The inhibition of bacterial RNAP is bactericidal. Therefore, we checked whether the growth inhibition also led to a killing of the bacteria via recovery assay. We analyzed recoverable chlamydiae to investigate the production of infectious EBs under different

CorA concentrations. In this assay we observed a significant reduction in infectious progeny of *C. trachomatis* serovars D and L2, *C. muridarum*, and *C. pneumoniae* treated with CorA in concentrations from 0.25 to 1.5 μg CorA (Figures 1D–F, Supplementary Figure 1A, and Supplementary Table 1) compared to the untreated control. The eradication rate was not significantly different between normoxia and hypoxia.

### CorA Eradicates Already Established Chlamydial Infections

**FIGURE 2:**
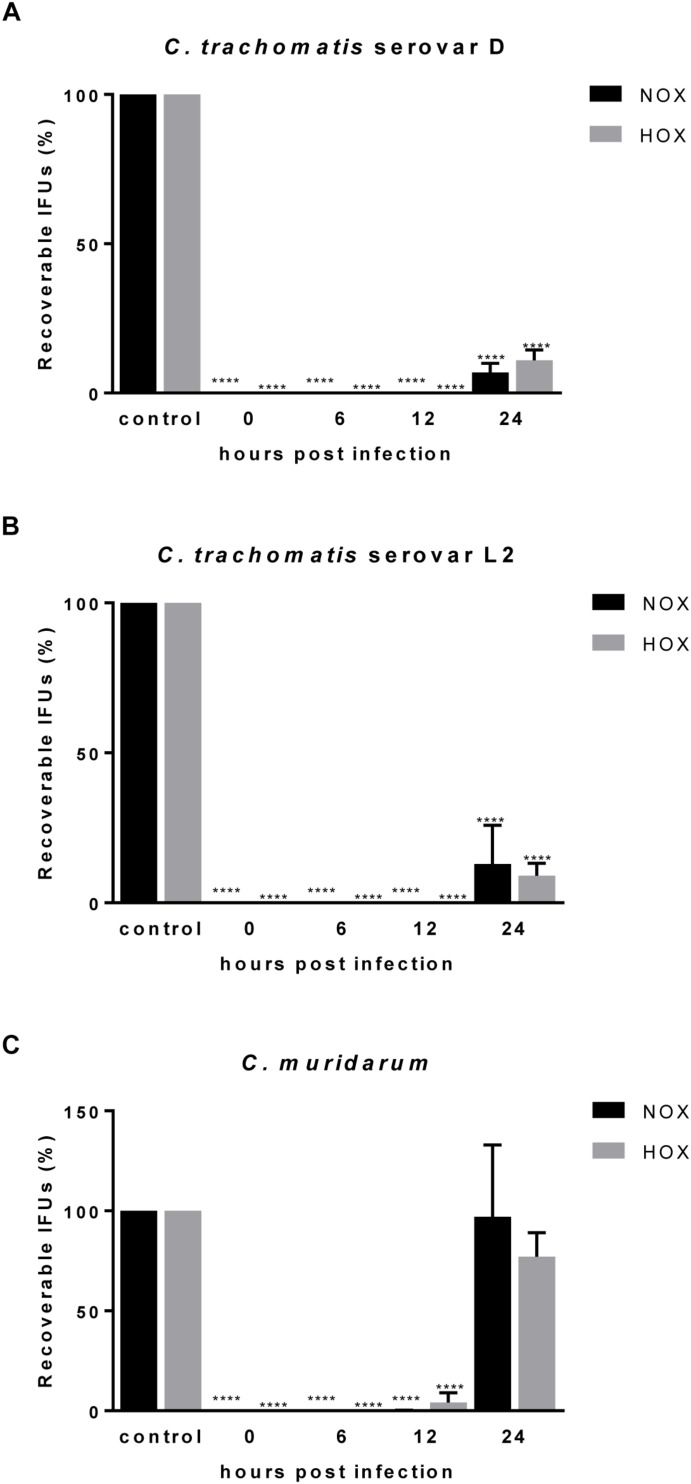
Efficacy of CorA against established *C. trachomatis* and *C. muridarum* infection in HeLa cells under normoxia and hypoxia. The eradication rate of *C. trachomatis* serovar D **(A)**, serovar L2 **(B)**, and *C. muridarum* **(C)** in HeLa cells. CorA (1 μg/mL) was added at 0, 6, 12, and 24 h.p.i. The numbers of recoverable chlamydiae in CorA treatment were calculated as a percentage of the untreated control under normoxia and hypoxia at 30 h.p.i. (*n* = 3; mean ± SD, Sidak’s multiple comparison ^****^p ≤ 0.0001). Nox: Normoxia, Hox: Hypoxia.

The majority of chlamydial infections has an asymptomatic course of infection and remains unnoticed for that reason. As a consequence, antimicrobial treatment is regularly initiated when the infection has already been long established. Therefore, we investigated whether CorA could effectively eradicate already established infections by delaying antimicrobial treatment throughout the chlamydial replication cycle for up to 24 h.p.i. When *Chlamydia* infected cells were treated with CorA (1.0 μg/mL) there was a significant reduction of infectious progeny for up to 12 h.p.i. in all tested strains. In *C. trachomatis* serovar D and serovar L2 as well as *C. pneumoniae* infected cells the reduction was still present if treated 24 h.p.i. (Figures 2A,B, Supplementary Figure 1B, and Supplementary Table 2), whereas the treatment 24 h.p.i. of *C. muridarum* infected cells led to chlamydial progeny comparable to the untreated control (Figure 2C and Supplementary Table 2). As in the previous experiments, no significant differences were observed between cells infected under normoxia and hypoxia.

### Application of CorA Showed No Impact on a *Chlamydia muridarum* Infection *in vivo*

**FIGURE 3:**
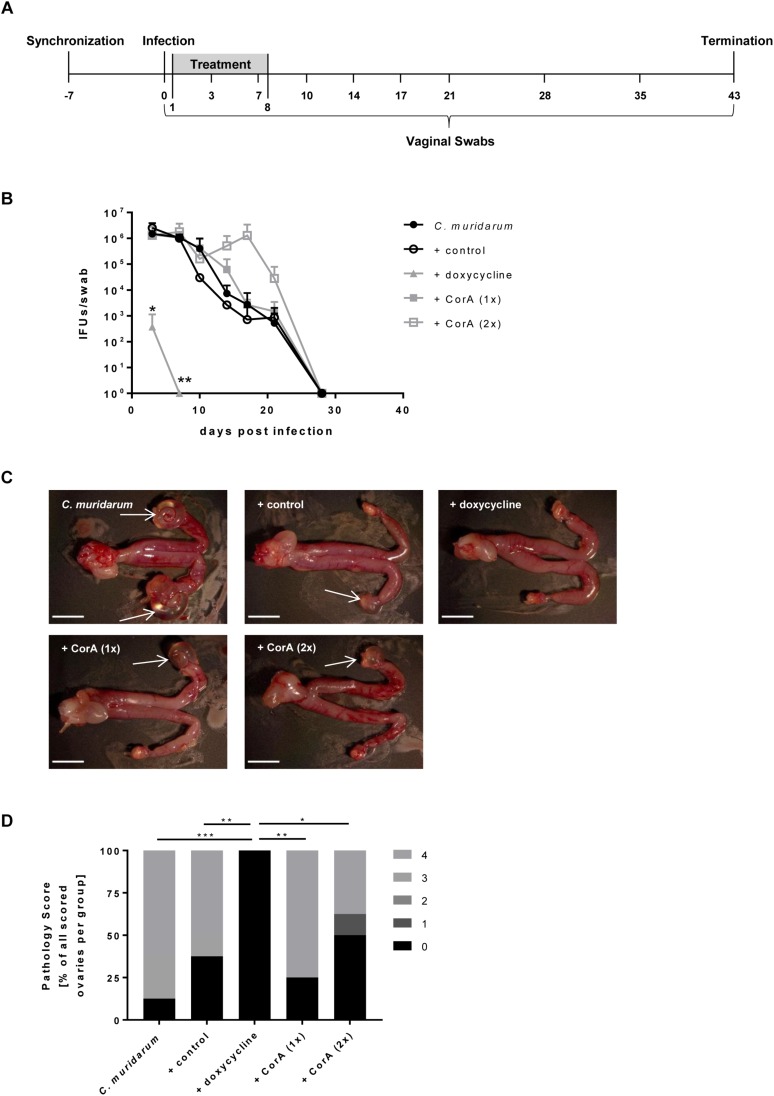
Efficacy of CorA against *C. muridarum in vivo*. Experimental setup of all conducted mouse experiments including sampling time points **(A)**. Bacterial shedding of *C. muridarum* in comparison to different treatments calculated from infectious progeny from vaginal swabs. (*n* = 4, Pairwise Wilcoxon rank sum test *p < 0.05, **p < 0.01) **(B)**. Formation of characteristic pathologies following infection with *C. muridarum* and treatment with corresponding substances (white arrows = hydrosalpinx, scale bar = 5 mm) **(C)**. Percentage of the observed pathology scores for all groups of mice (*n* = 8, Chi-Square test *p ≤ 0.05, **p ≤ 0.01, ***p ≤ 0.001) **(D)**. CorA: Corallopyronin A.

The usage of various animal models in chlamydial infection is broadly accepted, and has successfully been applied in investigations on CorA efficacy against other intracellular bacteria in mice (Schiefer et al., 2012; Kock et al., 2018). Hence, we were interested in the outcome of CorA treatment in an acute *C. muridarum* mouse infection model in comparison to standard doxycycline treatment. A general overview about the animal experiments is provided in Figure 3A. Oral administration of the anti-chlamydial antibiotics doxycycline, azithromycin, and amoxicillin was tested, and showed that doxycycline and azithromycin prevent hydrosalpinx formation and doxycycline eradicates *C. muridarum* completely (Supplementary Figure 2). Applying CorA either i.p. or orally, we analyzed bacterial shedding and hydrosalpinx formation during the course of infection. The bacterial burden 3 d.p.i. was comparable between all groups, while only the doxycycline treated mice showed a significantly reduced bacterial burden (Figure 3B and Supplementary Figure 2). Over time, control mice naturally shed *C. muridarum* over 28 days, with no recoverable pathogen on subsequent days (Figure 3B). *C. muridarum* was not detectable in doxycycline treated mice from 6 d.p.i. However, i.p. or orally CorA treated mice had a comparable shedding to the control mice (Figure 3B and Supplementary Figure 3A). Mice infected with *C. muridarum* developed hydrosalpinx on the ovaries with a mean score of 3.25 (Figures 3C,D). Neither the control mice (mean: 2.38) nor the mice treated with CorA (means: 3 and 1.63, respectively, CorA 1x or 2x) showed significant deviations from this pathology score (Figures 3C,D). In contrast, doxycycline completely averted the formation of hydrosalpinx (Figure 3D and Supplementary Figure 3B).

### CorA Inhibits the Growth of *C. trachomatis* Serovar D in an *ex vivo* Fallopian Tube Model

**FIGURE 4:**
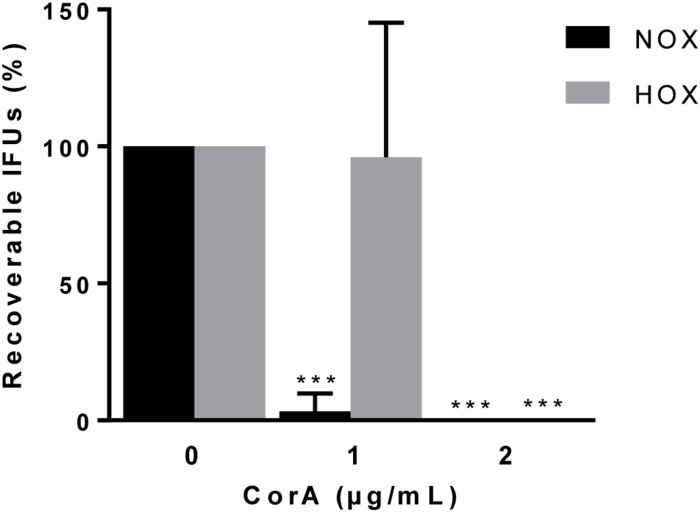
Efficacy of CorA against *C. trachomatis* serovar D in *ex vivo* culture of primary human fallopian tubes under normoxia and hypoxia. The inhibition rate of CorA on *C. trachomatis* serovar D growth under normoxia and hypoxia. The numbers of recoverable *C. trachomatis* serovar D under CorA treatment were calculated as a percentage of the untreated control at 48 h.p.i. (Normoxia *n* = 5; mean ± SD, Hypoxia *n* = 4; mean ± SD, Sidak’s multiple comparison ***p ≤ 0.001). Nox: Normoxia, Hox: Hypoxia, CorA: Corallopyronin A.

We aimed to test the efficacy of CorA against *C. trachomatis* as close as possible to the human *in vivo* setting. Therefore, we applied an *ex vivo* fallopian tube model that has been previously established for the analysis of host-pathogen interactions (Jerchel et al., 2012). Initially, we checked whether hypoxia affects the infectious outcome. We found that equal amounts of *C. trachomatis* could be recovered from infected fallopian tubes under normoxia and hypoxia (Supplementary Figure 4). We then tested whether CorA effectively inhibits the growth of *C. trachomatis* in this model. At 1 μg/mL CorA 2 ± 5% (normoxia) and 77 ± 60% (hypoxia) of *C. trachomatis* serovar D survived (Figure 4 and Supplementary Table 3). However, when we treated with 2 μg/mL CorA, growth of *C. trachomatis* serovar D was completely blocked in the *ex vivo* fallopian tube model under normoxia and hypoxia (Figure 4 and Supplementary Table 3).

## Discussion

More than 70% of *C. trachomatis* infections are asymptomatic, bearing the risk for progression during the acute infection phase or chronically over years (Molano et al., 2005). If appropriate therapy is not given or treatment is delayed in infected women, the pathogen may ascend from the lower female genital tract to the upper genital tract causing salpingitis and PID. Both are known risk factors for developing fallopian tube scarring, potentially leading to ectopic pregnancy and tubal infertility (Briceag et al., 2015; Hafner, 2015).

Doxycycline (100 mg orally twice a day for 7 days) or azithromycin (1 g orally in a single dose) are first line drugs to treat urogenital chlamydial infections and treatment is successful in >95% (Lau and Qureshi, 2002; Workowski et al., 2015). However, for longer periods following application of either doxycycline or azithromycin, 8% treatment failure has been observed (Fortenberry et al., 1999; Golden et al., 2005). Reduced intracellular activities of doxycycline and azithromycin were demonstrated under hypoxic conditions (Shima et al., 2011, 2013) and oxygen concentrations range from 0.5 to 5.5% in the cervix and vagina (Juul et al., 2007; Dietz et al., 2011). We therefore hypothesized that this is one of the possible reasons accounting for the insufficient eradication of *C. trachomatis* in the urogenital tract. Accordingly, we here present data on an alternative antimicrobial, CorA, with focus on its efficacy against intracellular *C. trachomatis* infection in cell culture and a human fallopian tube model, both under different oxygen environments and *in vivo*.

CorA binds to the bacterial RNAP, a highly conserved haloenzyme in bacteria with low homology to RNAP in eukaryotic cells. Thus, this antimicrobial is specific to prokaryotic RNAP (Irschik et al., 1985; Campbell et al., 2001; Belogurov et al., 2009). While CorA was originally described to inhibit the growth of Gram-positive bacteria (MIC value: between 0.1 and 10 μg/mL) (Irschik et al., 1985), it has recently been shown that it is also highly active against Gram-negative intracellular *O. tsutsugamushi* (Kock et al., 2018) and *Wolbachia* endobacteria *in vitro* and *in vivo* (Schiefer et al., 2012). The structure and preponderance of efflux pumps of many Gram-negative bacteria seems to be a barrier to CorA (Irschik et al., 1985). Therefore, it was proposed that it might be effective against *Wolbachia* because they are not able to synthesize lipopolysaccharides (Schiefer et al., 2012). Chlamydial LPS has a unique and atypical structure of its lipid A (Kosma, 1999) and lacks O-antigen polysaccharide side chains (Rund et al., 1999). Further, *Chlamydia* do not encode for TolC, an outer membrane tunnel of multidrug resistance efflux pumps that has been implicated in CorA export in *E. coli* (Mariner et al., 2011). Therefore, we hypothesized that CorA might also be effective against obligate intracellular chlamydiae.

In this study, MICs against *C. trachomatis* serovars D and L2 were 0.5 μg/mL under normoxia and hypoxia, while recovery of infectious EBs was limited already at 0.25 μg/mL. Using recovery assays, we could further show strong CorA activity against chlamydiae during an established infection under both normoxia and hypoxia. Our data indicate that CorA has a high efficacy against *C. trachomatis*, wherein the activity of CorA appears not to be influenced by the environmental oxygen availability in cell culture, an important finding given the described impact of oxygen on standardly prescribed antibiotics (Shima et al., 2011, 2013). Furthermore, we have already described CorA as a potent substance against rifampin-resistant *C. trachomatis* L2 serovars (Shima et al., 2018), thereby enhancing the application area of CorA. We were curious whether efficacy is also observed against other pathogenic *Chlamydia* and observed that CorA inhibits *C. muridarum* in a similar concentration, while *C. pneumoniae* CWL029 is inhibited at a MIC of 1 μg/mL (Table 1 and Supplementary Figure 1). This indicates a general activity of CorA across the *Chlamydia* genus. While treatment throughout the first hours of the infection has equal efficacies, we observed enhanced chlamydial recovery if treated after 24 h for *C. muridarum* (Figure 2), indicating that CorA acts mainly against the replicative reticulate bodies of the chlamydial cell cycle. In favor of this assumption, no increase in recovery is observed if treatment takes place 24 h.p.i. in *C. pneumoniae* infection (Supplementary Figure 1B), reflecting a longer duration of its life cycle and, thus, later redifferentiation to elementary bodies.

We tested the impact of CorA in an *in vivo* model against the murine pathogen *C. muridarum*, as it successfully infects the genital tract of female mice (Barron et al., 1981). Since the MIC against several chlamydial species is comparable to the MIC of CorA against *Wolbachia*, our mouse experiments used the same doses or even double of published CorA doses (Schiefer et al., 2012). In these experiments, CorA has been shown to effectively target *Wolbachia*, essential intracellular endosymbionts of filarial nematodes, after intraperitoneal administration (Schiefer et al., 2012).

Although doxycycline was able to eradicate *C. muridarum* in our *in vivo* model, no comparable effect was achieved when CorA was administered, neither i.p. nor orally. As with amoxicillin, the resulting hydrosalpinx were comparable to infected control animals. A wide range of factors need to be considered when addressing the *in vivo* results. Thus, in contrast to the human pathogens *C. trachomatis* and *C. pneumoniae*, the CorA treatment that was initiated 24 h after infection was also not sufficient in depleting the murine pathogen *C. muridarum in vitro* (Figure 2C). The start of the treatment in the *in vivo* model mimics the same conditions, by infecting the mice and starting the treatment 24 h after infection. Thus, the timing of the treatment in conjunction with strain-specific life cycle kinetics may contribute to the missing *in vivo* activity of CorA.

Further reasons for the lack of treatment success in our mouse model may be partially attributed to differences in available treatment settings compared to studies with other pathogens. While successful *Wolbachia* treatment is performed over a course of 2–4 weeks (Schiefer et al., 2012; Schiefer et al., unpublished data), resulting in a long lasting antibiotic pressure on the bacteria, this cannot be performed in the short, but intensive infection with *C. muridarum*. In the case of the treatment of *O. tsutsugamushi* the treatment duration is comparable to our setting. However, treatment was applied directly at the site of infection (Kock et al., 2018) and is thus not comparable to our model.

While CorA availability in the plasma of mice has been shown earlier (Shima et al., 2018), impaired drug penetration to the infected tissues is a problem in urogenital diseases (Muller et al., 2004). Thus, a short duration of high plasma levels of CorA in combination with limited drug penetration may eventually lead to a blood-tissue disequilibrium preventing successful treatment of *C. muridarum* infection in the mouse. This may have been exacerbated by the formulation used. Although CorA dosages were adjusted taking into account the 60% purity of CorA, a liquid formulation able to fully dissolve CorA at high concentrations and suitable for *in vivo* experiments is needed to achieve good genital tissue penetration.

While *in vivo* treatment of *C. muridarum* infection in the mouse is hampered during classical treatment regimens (e.g., oral administration), future experiments could make use of other application strategies, e.g., direct administration of CorA to the vagina. Local administration of metronidazole as gels and tablets is commonly used in the clinics for the treatment of bacterial vaginosis in females (Menard, 2011), but no models exist for such treatment of STIs.

Severe pathological sequels of *C. trachomatis* infections of the female genital tract frequently take place in the fallopian tubes. These are muscular conduits connecting the ovaries with the uterus and therefore play a key role for fertilization and transport of the developing blastocyst to the uterus. *C. trachomatis* infection causes salpingitis, which leads to functional damage of the fallopian tubes (Hafner, 2015). Explanted fallopian tubes have been shown to provide a powerful tool for the investigation of chlamydial infection (Roth et al., 2010; Jerchel et al., 2012; Kessler et al., 2012). As the whole organ is infected *in vivo* in ascending genital infections, our *ex vivo* system provides a model that mirrors the *in vivo* human state, and provides additional value in performing infection experiments with *C. trachomatis* in comparison to human cell lines and experimental animal models (Cooper et al., 1990; Jerchel et al., 2012; Kessler et al., 2012). In this study, we could show that the growth of *C. trachomatis* was completely blocked by 2 μg/mL CorA treatment in the fallopian tube *ex vivo* model under hypoxia, while 1 μg/mL was sufficient under normoxia. This difference in effectivity might be related to the complexity of the tissue structure of the fallopian tube and diverging behavior compared to a monolayer cell culture. As CorA is effective in reasonable doses in fallopian tubes, it might have the potential to eradicate *C. trachomatis* not only in cell-based approaches, but also *in vivo* in genital infection in humans as such.

Taken together, our data indicate that CorA is highly active against *C. trachomatis* in cervical epithelial cells and human fallopian tubes, under normoxia as well as hypoxia. This provides an advantage over currently used antibiotics that have been shown to lose efficacy under normal physiologic oxygen tension. Thus, CorA should be further developed as an *in vivo* treatment option for chlamydial infections. In addition, there is a range of anti-bacterial compounds produced from myxobacteria besides CorA (Schaberle et al., 2014; Herrmann et al., 2017) including several derivates of CorA (Schaberle et al., 2015), which should be screened for their efficacy against *Chlamydia*.

## Author Contributions

NL, SG, SL, KS, IK, FH, and BH performed the experiments. JR, SG, NL, AH, KP, AS, BH, and IK contributed to the conception of the work. NL, SG, and KS performed statistical analysis. NL, SG, KS, and JR prepared the first draft of the manuscript. All authors read and approved the final version of the manuscript.

## Conflict of Interest Statement

AH, AS, and KP hold patents for Corallopyronin A in the United States and European Union (US 9168244 B2, US 9687470 B2, and EP 2704708 B1). The remaining authors declare that the research was conducted in the absence of any commercial or financial relationships that could be construed as a potential conflict of interest.
