# Supplementary figures and images for: Elaborations on Corallopyronin A as a Novel Treatment Strategy Against Genital Chlamydial Infections
Source: Front Microbiol. 2019 May 7;10:943. doi: 10.3389/fmicb.2019.00943 (PMC6514060; doi:10.3389/fmicb.2019.00943)

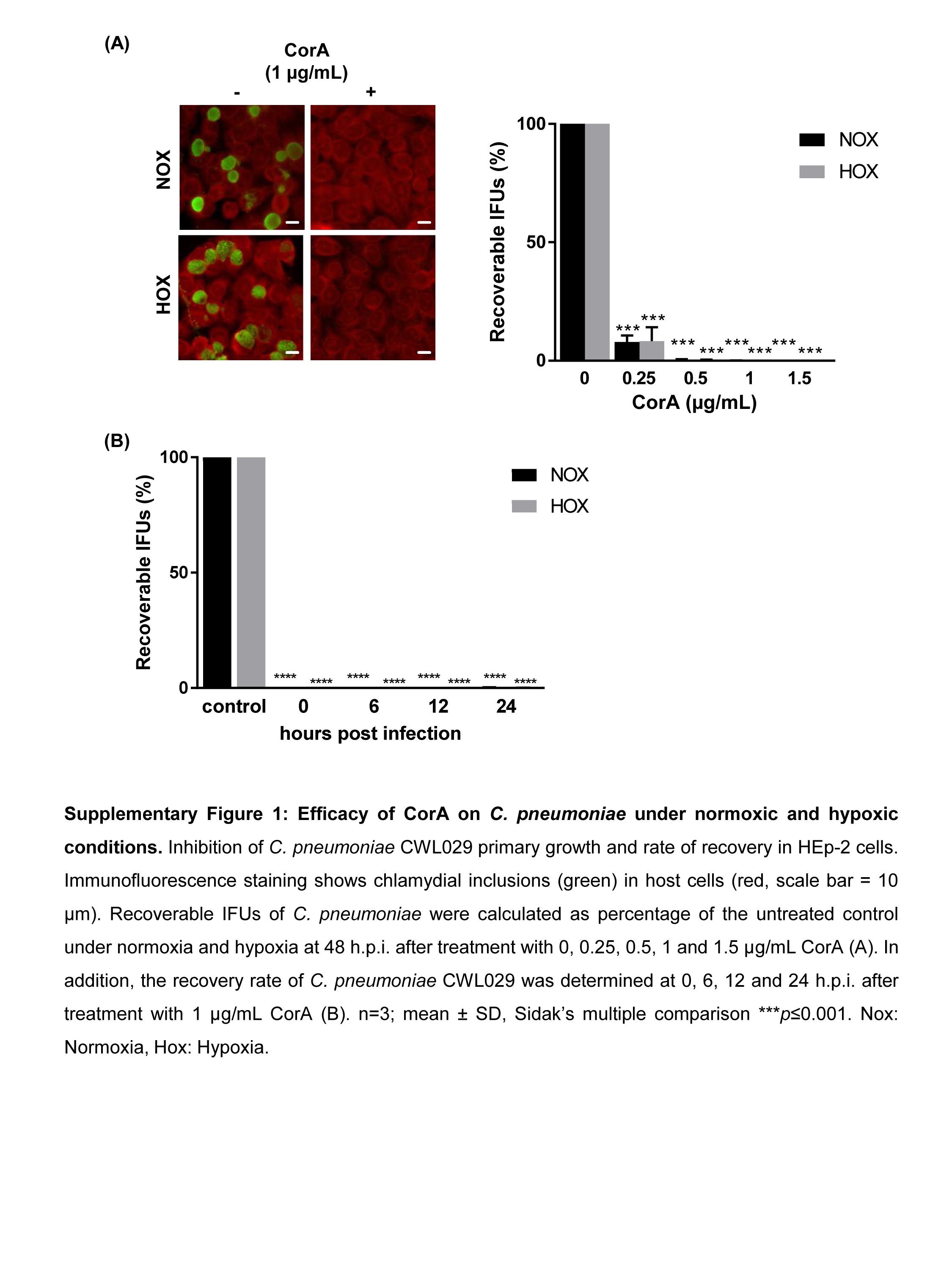

Supplement: Supplementary file 1 [file Image_1.TIF]

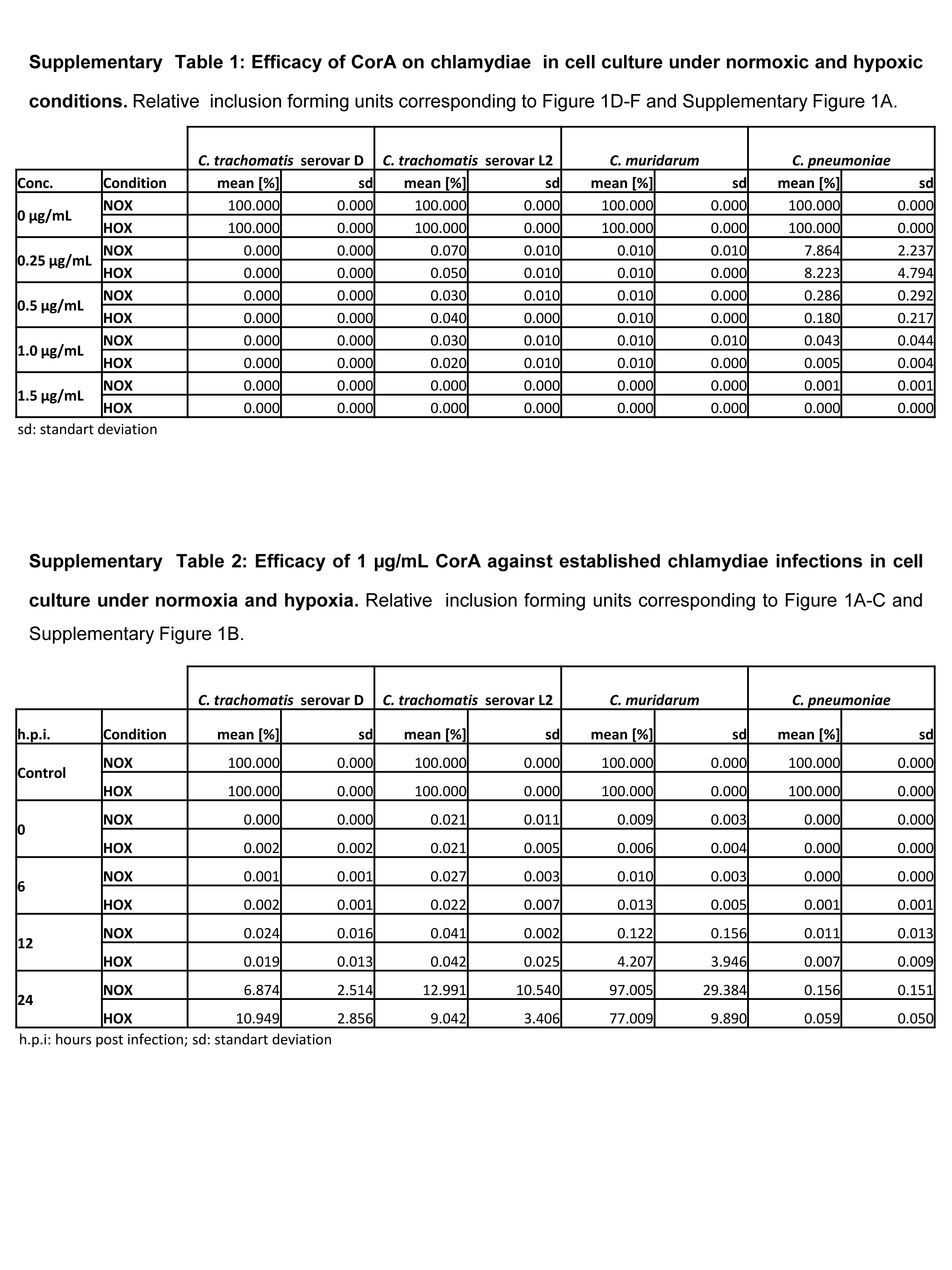

Supplement: Supplementary file 2 [file Image_2.TIF]

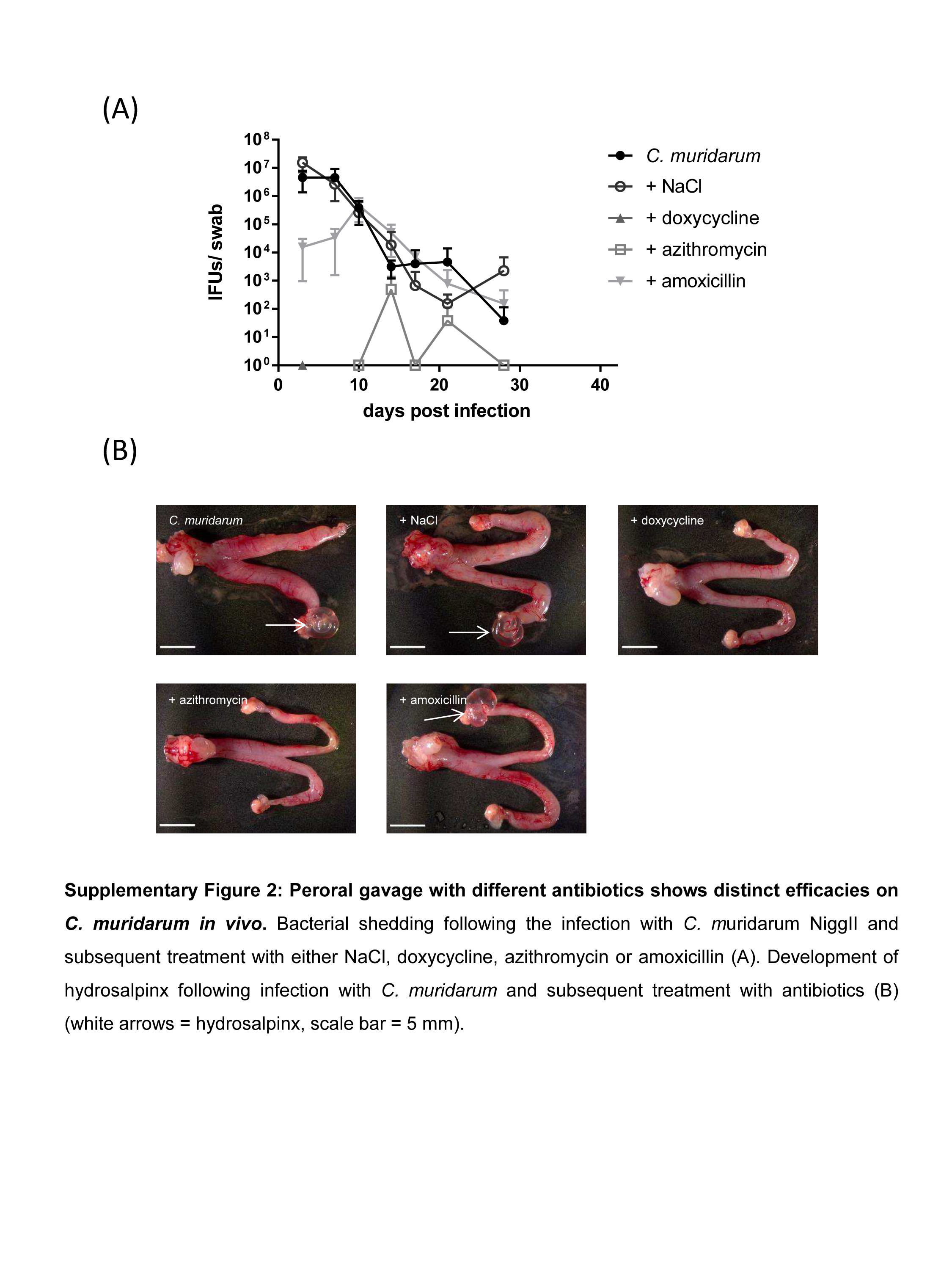

Supplement: Supplementary file 3 [file Image_3.TIF]

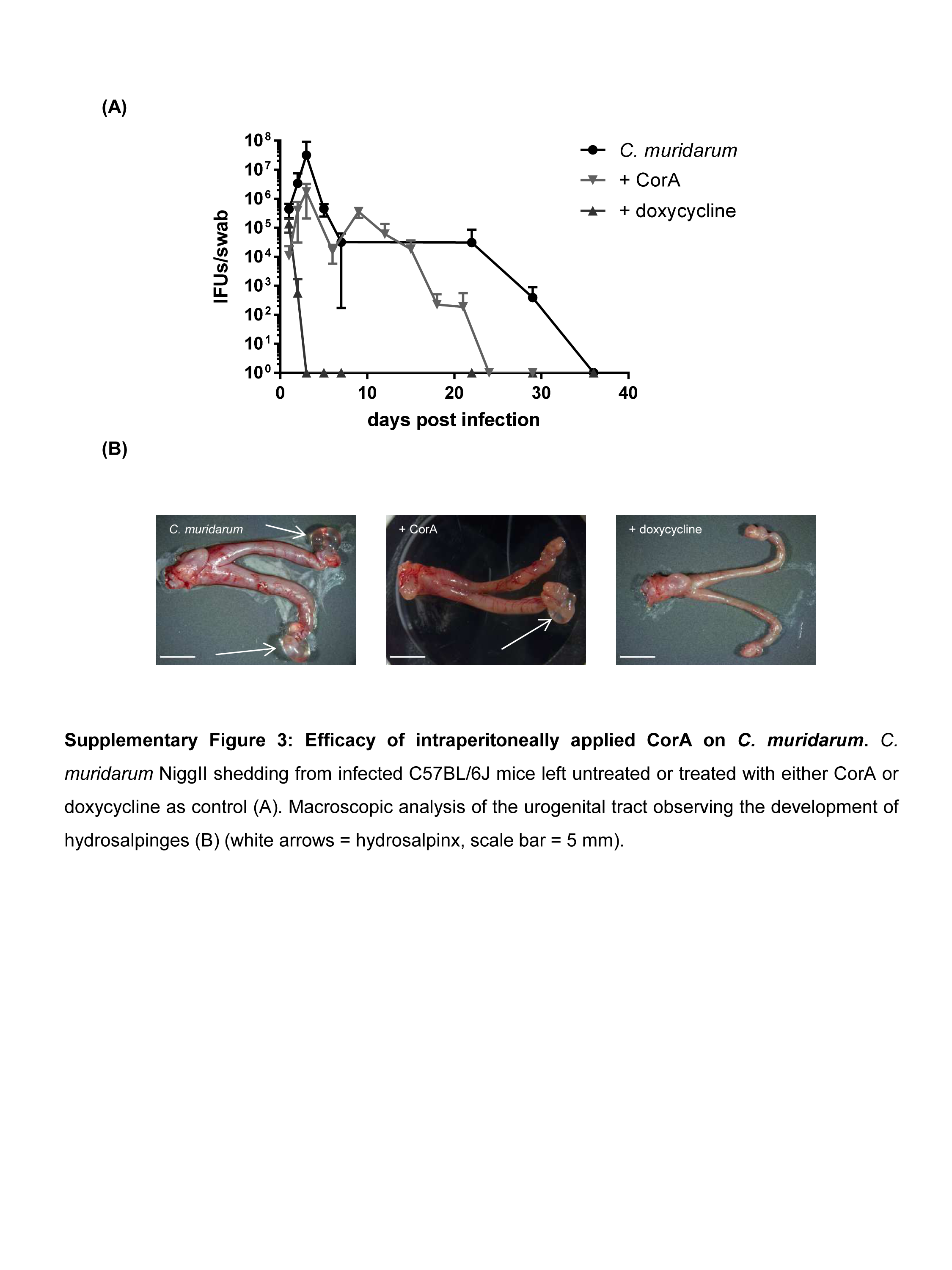

Supplement: Supplementary file 4 [file Image_4.TIF]

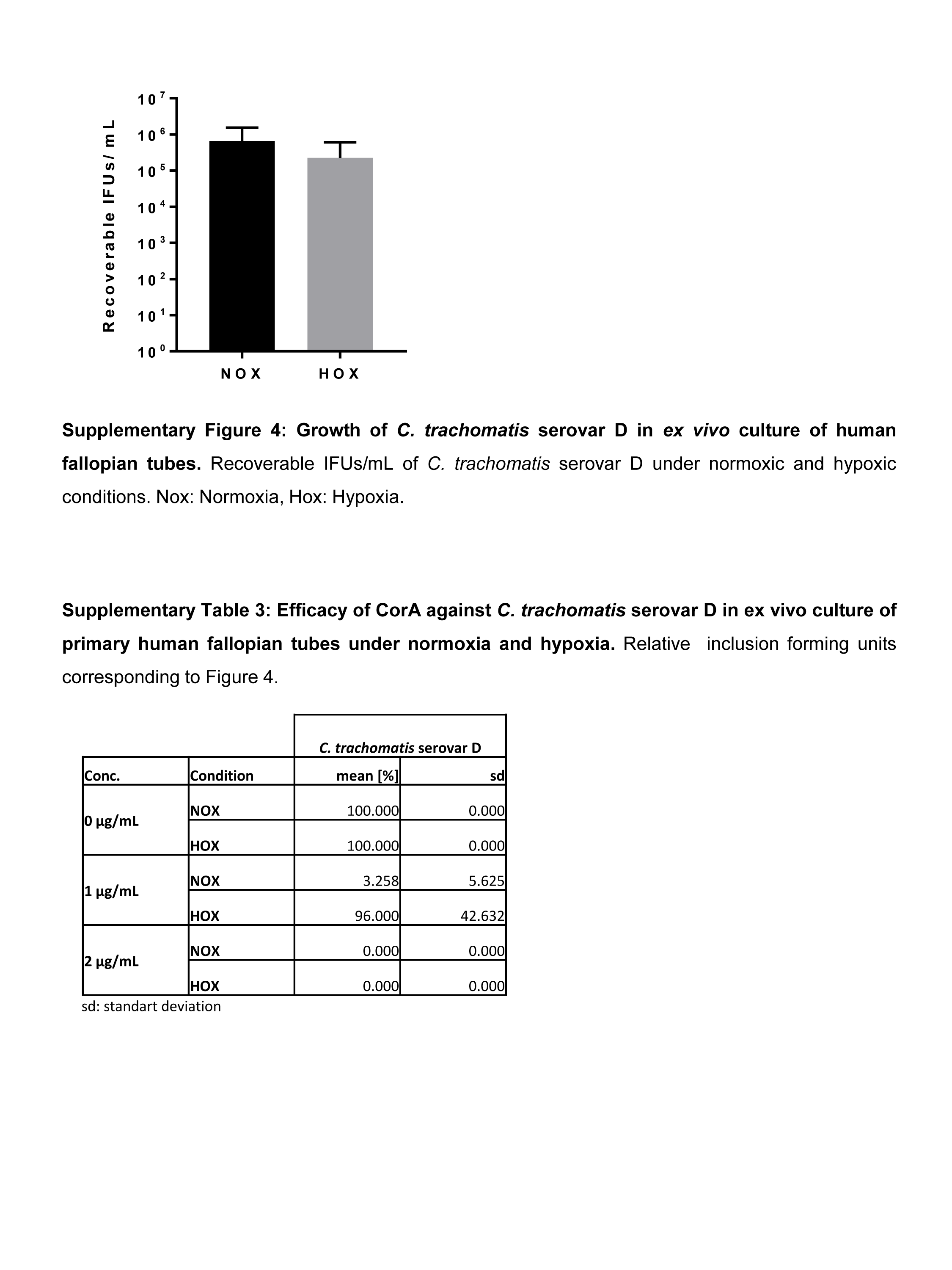

Supplement: Supplementary file 5 [file Image_5.TIF]
